# Machine learning and single-cell RNA sequencing analyses identify MS-related monocytes and a five-gene candidate biomarker signature

**DOI:** 10.3389/fneur.2026.1739231

**Published:** 2026-02-11

**Authors:** Di Pan, Xinyi Wei, Xiyan Kuang, Dan Yang

**Affiliations:** Department of Neurology, The Second Affiliated Hospital of Harbin Medical University, Harbin, China

**Keywords:** bioinformatics, experimental autoimmune encephalomyelitis (EAE), monocytes, multiple sclerosis, optimally characterized genes

## Abstract

**Objective:**

Multiple sclerosis (MS) is a chronic autoimmune inflammatory disease of the central nervous system (CNS). Based on single-cell RNA sequencing (scRNA-seq) data from experimental autoimmune encephalomyelitis (EAE), this study applied machine learning algorithms combined with integrative bioinformatics methods to identify pivotal biomarkers associated with MS-related monocytes.

**Materials and methods:**

Machine learning and scRNA-seq analyses were performed to characterize MS-related monocytes, leading to the identification of five optimally characterized candidate biomarkers associated with pathogenic alterations. The performance of multiple algorithms, such as logistic regression (LogReg), latent Dirichlet allocation (LDA), support vector machine (SVM), Naive Bayes (NB), k-nearest neighbor (KNN), Rpart, and random forest (RF), was evaluated. In addition, the CIBERSORT, single-sample gene set enrichment analysis (ssGSEA), and GSEA algorithms were employed to investigate and define immunological features and biological functions. Finally, quantitative real-time polymerase chain reaction (qRT-PCR) and immunofluorescence were used to validate the expression of the identified genes.

**Results:**

Seven machine learning algorithms consistently validated five key genes (*COX5A*, *CTSS*, *GBP2*, *IRF7*, and *PGAM1*) as optimally characterized biomarkers. The infiltration profiles of these genes, together with associated immune cell types, provide potential biological underpinnings for the pathogenic alterations observed in MS.

**Conclusion:**

Collectively, these findings indicate that COX5A, CTSS, GBP2, IRF7, and PGAM1 represent promising biomarkers for MS. The identified gene signature may improve MS diagnosis and risk stratification and provide new insights into monocyte-driven immunopathology.

## Introduction

Multiple sclerosis (MS) is a chronic disorder of the central nervous system (CNS) characterized by autoimmune-mediated demyelination of the myelin sheath (1). Axonal disruption in neuronal functional regions leads to recurrent episodes of symptom remission and relapse, observed in approximately 85% of patients with MS. The typical clinical presentation includes unilateral or bilateral limb weakness caused by transverse myelitis, along with sensory deficits or impairments. Demyelinating lesions in the brainstem or cerebellum can manifest as diplopia, brainstem dysfunction, and cerebellar ataxia (2). Owing to its distinct histopathological features and relapsing clinical course, MS is considered a highly complex and heterogeneous neurological disease (3).

Genetic and environmental factors influencing myelin-specific CD4^+^ T cells play a central role in the disease’s pathophysiology, ultimately driving progressive neurodegeneration (4). Both innate and adaptive immune responses contribute to MS pathogenesis. Innate immune mechanisms disrupt the blood–brain barrier (BBB) and initiate inflammatory cascades, while imbalances among Th1, Th2, Treg, and Th17 cells are strongly associated with disease progression (5). Monocytes, which are also involved in adaptive immune responses (6), have been shown to play a key role in MS. Transcriptionally intermediate CD14^+^CD16^+^ monocytes (7) are rapidly activated to release inflammatory cytokines such as IL-6 and IL-12 in MS (8). These monocytes further promote the upregulation of miR-155 (9) and the co-stimulatory molecule CD80, thereby amplifying T-cell-mediated inflammatory responses. In addition, the presence of neutralizing anti-drug antibodies in MS patients receiving interferon-*β* therapy has been associated with increased CD14^+^ monocyte frequency (10). The experimental autoimmune encephalomyelitis (EAE) model, induced by immunization with myelin antigens, is widely used in MS research (11). In recent years, the brain–gut axis has also been implicated in immune-mediated diseases. Using the EAE model, Haghikia et al. (12) demonstrated that short-chain fatty acids modulate T-cell differentiation toward regulatory T cells while suppressing pro-inflammatory T lymphocytes, ultimately alleviating MS-related inflammation. Despite these advances, few studies have systematically investigated transcription factor (TF) regulatory networks and risk genes that contribute to MS pathogenesis.

Early initiation of disease-modifying therapies (DMTs) is recommended for MS patients with recurrent episodes and significant functional impairment. Immunosuppressive and immunomodulatory agents are the first-line DMTs, aimed at preventing relapses and limiting the development of new lesions (13). A comprehensive overview of available DMTs is provided by the Multiple Sclerosis Coalition^1^ (14). Despite their efficacy, current immune-modifying therapies have important limitations. For example, natalizumab, which prevents activated T cells from crossing the BBB by inhibiting the α4-integrin adhesion molecule, has been shown to reduce relapse rates by 68% and disability progression by 40% (15). However, profound immunodeficiency resulting from long-term immunosuppression decreases CD4^+^ T-cell counts. Extended natalizumab treatment has also been associated with reactivation of the John Cunningham virus, leading to progressive multifocal leukoencephalopathy (16). These challenges highlight the urgent need for identifying genetic predispositions and critical molecular targets that could guide novel therapeutic strategies.

In this study, we applied the HdWGCNA pipeline to identify synergistically co-expressed gene sets in MS-related monocyte subclusters. Machine learning algorithms were then integrated with scRNA-seq data to predict MS risk. In parallel, we employed bioinformatics approaches to characterize immunological features, regulatory networks, and potential biomarker relevance of key genes. Collectively, these findings suggest that the identified biomarkers may offer new opportunities for MS diagnosis and prognosis. Such biomarkers have the potential to improve clinical outcomes by enabling earlier prevention strategies and reducing disease burden.

## Materials and methods

### Data collection and preprocessing

All transcriptomic data used in this study were obtained from the Gene Expression Omnibus (GEO) database.^2^ This included the single-cell RNA sequencing dataset GSE199460 (comprising 6 samples), along with two bulk microarray datasets: GSE135511 (GPL6883 platform; 10 controls and 40 MS samples) and GSE108000 (GPL13497 platform; 10 controls and 30 MS samples). Single-cell sequencing data were processed using the Seurat package (version 4.2) (17). Cells were retained if they expressed between 200 and 5,000 genes (nFeature_RNA) and contained less than 20% mitochondrial transcripts (Supplementary Figures S1A,B). Principal component analysis (PCA) was performed on highly variable genes (Supplementary Figure S1C). To mitigate batch effects, data integration was carried out using the Harmony algorithm with default parameters (theta = 2; lambda = 1), grouping cells by sample origin (group.by.vars = “orig.ident”; Supplementary Figure S1D). The top 20 principal components from the harmonized data were used for non-linear dimensionality reduction via Uniform Manifold Approximation and Projection (UMAP; Supplementary Figure S1E). Unsupervised cell clustering was performed on the UMAP embedding using the ‘FindClusters’ function at a resolution of 0.6. Cell types were annotated automatically with the SingleR package and subsequently validated by inspecting the expression of canonical lineage-specific marker genes (18), and the number of cells in each category was quantified. We performed validation of the canonical monocyte protein markers (Supplementary Figure S2).

### Cellular communication analysis

Cell–cell communication analysis (CellChat package v1.0.0) was employed to examine the interactions of MS-related monocytes (19). This approach reconstructs intercellular signaling networks using differentially expressed ligand–receptor gene pairs. The statistical significance of each inferred interaction was evaluated by permutation testing, and only interactions with an adjusted *p*-value below 0.05 were retained for further analysis (20).

### High-dimensional weighted gene co-expression network analysis (WGCNA)

WGCNA is a powerful method for identifying gene expression patterns across biological processes. The HdWGCNA package (version 0.1.1.9010) extends this approach for scRNA-seq data, providing a high degree of modularity (21). Co-expression modules associated with MS-related monocyte subsets were identified. Module eigengenes were calculated to represent the overall expression pattern of each module. Hub genes within a module were defined as the top 10 genes exhibiting the highest connectivity to the module eigengene, as measured by the module membership value (kME). Genes with a kME greater than 0.8 were typically considered strongly connected and selected as hub genes.

### Pseudotime analysis

Pseudotemporal trajectories of monocytes were analyzed using the Monocle2 algorithm. Prior to trajectory construction, marker genes were selected based on clustering results, and primitive cell expression counts were screened for analysis (22). This pseudotime analysis enabled the characterization of key dynamic changes in hub genes associated with MS-related monocyte subpopulations during monocyte maturation.

### Differential expression analysis

Bulk RNA-seq analysis identified a total of 55 differentially expressed genes (DEGs). Differential expression between MS and control groups was assessed using the “limma” R package, with the thresholds set at |logFC| > 0.5 and *p* < 0.05. Volcano plots and heatmaps were subsequently generated to visualize significantly upregulated and downregulated genes.

### Functional enrichment analysis

To investigate the biological functions of DEGs, enrichment analyses were performed using Gene Ontology (GO), Kyoto Encyclopedia of Genes and Genomes (KEGG), and Disease Ontology (DO) via the “clusterProfiler” and “DOSE” R packages. In addition, protein–protein interaction (PPI) networks were constructed using the STRING database (23) to evaluate interactions among overlapping genes. Co-expression networks were further visualized with the “igraph” R package based on correlation strengths.

### Construction of machine learning models

Machine learning models were constructed to screen and validate optimal biomarker candidate genes. Optimally characterized genes were filtered from the DEGs using the least absolute shrinkage and selection operator (LASSO) regression combined with 10-fold cross-validation (24). Seven machine learning models, logistic regression (LogReg), linear discriminant analysis (LDA), support vector machine (SVM), Naive Bayes (NB), k-nearest neighbor (KNN), recursive partitioning (Rpart), and random forest (RF), were constructed based on the optimally characterized genes using the mlr3verse package (version 0.2.7). These models were applied to establish predictive frameworks for gene expression data, enabling the identification of the most suitable algorithm and key candidate biomarkers.

### Expression patterns and biomarker relevance of optimally characterized genes

The expression levels of optimally characterized genes were assessed in both the training dataset (GSE135511) and the validation dataset (GSE108000). Statistical analyses were performed using the *t*-test for normally distributed data and the Wilcoxon rank-sum test for non-parametric comparisons. Discriminative ability was evaluated by constructing receiver operating characteristic (ROC) curves with the pROC R package (25), and the area under the curve (AUC) was calculated to quantify predictive accuracy.

### Single-cell regulatory network inference and clustering (SCENIC) analysis of TF regulatory networks

SCENIC (version 1.2.4) (26) was applied to identify TF-driven gene regulatory networks and cellular states from single-cell sequencing data. The SCENIC algorithm was used to investigate differential TF activity between MS and control samples and to evaluate the associations between optimally characterized genes and specific TFs.

### Immune infiltration analysis

Immune infiltration analysis was conducted with the objective of correlating molecular discoveries with alterations in the immune microenvironment. The CIBERSORT algorithm, developed by Newman et al. (27), was applied to estimate the relative proportions of 22 immune cell types within mixed cell populations. Using the GSE135511 dataset, immune cell infiltration levels were quantified for MS and control samples. Differences in immune cell proportions between groups were evaluated using the Wilcoxon rank-sum test.

### Assessment of hallmark gene sets

To assess the reactivation patterns of optimally characterized genes, the single-sample gene set enrichment analysis (ssGSEA) algorithm, implemented via the GSVA package (version 1.42.0), was applied to quantify the relative enrichment scores of 50 hallmark gene sets (h.all.v7.5.1.symbols.gmt) in the GSE135511 dataset (28). This analysis revealed the signaling pathways associated with optimally characterized genes.

### GSEA and correlation analysis of optimally characterized genes

To further explore the biological functions of MS-associated DEGs, gene set enrichment analysis (GSEA) was performed to identify enriched pathways. Gene enrichment was evaluated separately for high- and low-expression groups. Finally, Pearson correlation analysis was conducted to assess the relationships among optimally characterized genes.

### Animals

Female C57BL/6J mice (10–12 weeks old) were used in this study. All experiments were performed in accordance with guidelines and protocols approved by the Animal Research Committee of Harbin Medical University (Approval No. SYDW2020-047). Mice were housed under specific pathogen-free (SPF) conditions in the Experimental Animal Center of the Second Affiliated Hospital of Harbin Medical University. Animals were maintained on a 12-h light/dark cycle with ad libitum access to food and water. All procedures were conducted in SPF barrier facilities under standard operating protocols.

### Active immunization for EAE induction

EAE was induced by active immunization. Mice were briefly anesthetized with 2% isoflurane and subcutaneously injected with 200 μg of MOG_35-55_ peptide emulsified in complete Freund’s adjuvant (CFA) at the groin and lower back. Pertussis toxin (400 ng) was administered intravenously via the tail vein at 2 h and 24 h after immunization. From day 0 to day 22 post-injection (dpi), mice were monitored daily for neurological symptoms, and clinical scores were assigned to evaluate physical disability. The scoring system was as follows: 0. No clinical signs; 1. Limp tail; 2. Unsteady gait with partial hind limb weakness; 3. Complete hind limb paralysis; 4. Paralysis of all four limbs; and 5. Moribund or death. All mice were sacrificed at 22 dpi.

### Quantitative real-time polymerase chain reaction (qRT-PCR)

Mice were anesthetized and perfused with cold PBS. Brain tissues were collected, fixed in 4% paraformaldehyde, embedded in paraffin, and sectioned into 20-μm transverse slices. Total RNA was extracted using TRIzol™ reagent (Invitrogen, Carlsbad, CA, USA). RNA was reverse-transcribed into cDNA with the SuperScript™ One-Step Reverse Transcription Kit (Invitrogen) according to the manufacturer’s instructions. Gene expression levels of *COX5A*, *CTSS*, *GBP2*, *IRF7*, *PGAM1*, and the internal control *β*-actin were quantified by qRT-PCR on an Applied Biosystems platform (USA). Relative expression levels were calculated using the comparative threshold cycle (2^-∆∆Ct^) method.

IRF7 primer: forward, 5’-CTGCTTTCTGGTGATGCTGG-3′ and reverse, 5’-GTAGCTTCCATCTGCCATGC-3′; COX5A primer: forward, 5’-GTTGGACCAATCATAGGCGCT-3’and reverse, 5’-CAATGTCGATCACATGCACCA-3′; PGAM1 primer: forward, 5’-TGCATACCTGCGATCTATTGCACATCACTC-3’and reverse, 5’-CACTGATCTACCGTATTTGCTGT-3′; CTSS primer: forward, 5’-CACGCAGAACGTGAACACC-3′, and reverse, 5’-GGCAGTAGATAACGTGAGGGA-3′; GBP2 primer: forward, 5’-ACACCAACAAGTAACGATGCC-3′, and reverse, 5’-GCAAAGGTTTCACTTTCCCCA-3′; *β*-actin primer: forward, 5’-AAGTCCCTCACCCTCCCAAAAG-3′, and reverse, 5’-AAGCAATGCTGTCACCTTCCC-3′.

### Immunofluorescence staining

Immunofluorescence was performed to detect the localization of COX5A, CTSS, GBP2, IRF7, and PGAM1 expression in brain tissue. Coronal brain sections (20 μm) were prepared using a frozen microtome (Leica CM1900, Wetzlar, Hesse, Germany) and mounted on slides. Sections were incubated at 4 °C for 24 h with the following primary antibodies: anti-COX5A (mouse, Abcam, ab180129, 1:100), anti-GBP2 (rabbit/mouse, Santa Cruz Biotechnology, 1:100), anti-CTSS (mouse, Santa Cruz, sc-271619, 1:50), anti-IRF7 (mouse, Santa Cruz, sc-74471, 1:200), anti-PGAM1 (mouse, Santa Cruz Biotechnology, 1:100), and anti-CD11b (rat, clone M1/70.15, AbD Serotec, Kidlington, UK, 1:200). After washing, sections were incubated with fluorescent secondary antibodies for 4 h at 37 °C: Alexa Fluor 488-conjugated anti-rabbit IgG (green) or Alexa Fluor 594-conjugated anti-mouse IgG (red).

## Results

### Single-cell sequencing cluster analysis of EAE

Myelin oligodendrocyte glycoprotein (MOG_35-55_)-induced EAE is widely used to study the pathophysiology of MS and to evaluate potential therapeutic strategies. The pathological features of EAE closely resemble those observed in MS patients, such as inflammatory infiltration of brain tissue, axonal damage, and dysregulated immune responses (29). Unsupervised clustering of integrated single-cell transcriptomes identified 26 transcriptionally distinct cell clusters. Annotation based on the expression profiles of canonical lineage marker genes classified these clusters into microglia, monocytes, macrophages, T cells, B cells, astrocytes, oligodendrocytes, endothelial cells, epithelial cells, fibroblasts, and neurons (Supplementary Figure S1F). Comparative analysis of cellular composition between the EAE group and the control group revealed significant differences in proportions of multiple cell types, most notably in T cells, B cells, astrocytes, epithelial cells, and monocytes (Supplementary Figures S1G,H). Notably, monocytes exhibited the most pronounced expansion within the EAE group (Supplementary Figure S1I).

### Analysis of MS-associated cellular communication

**Figure 1:**
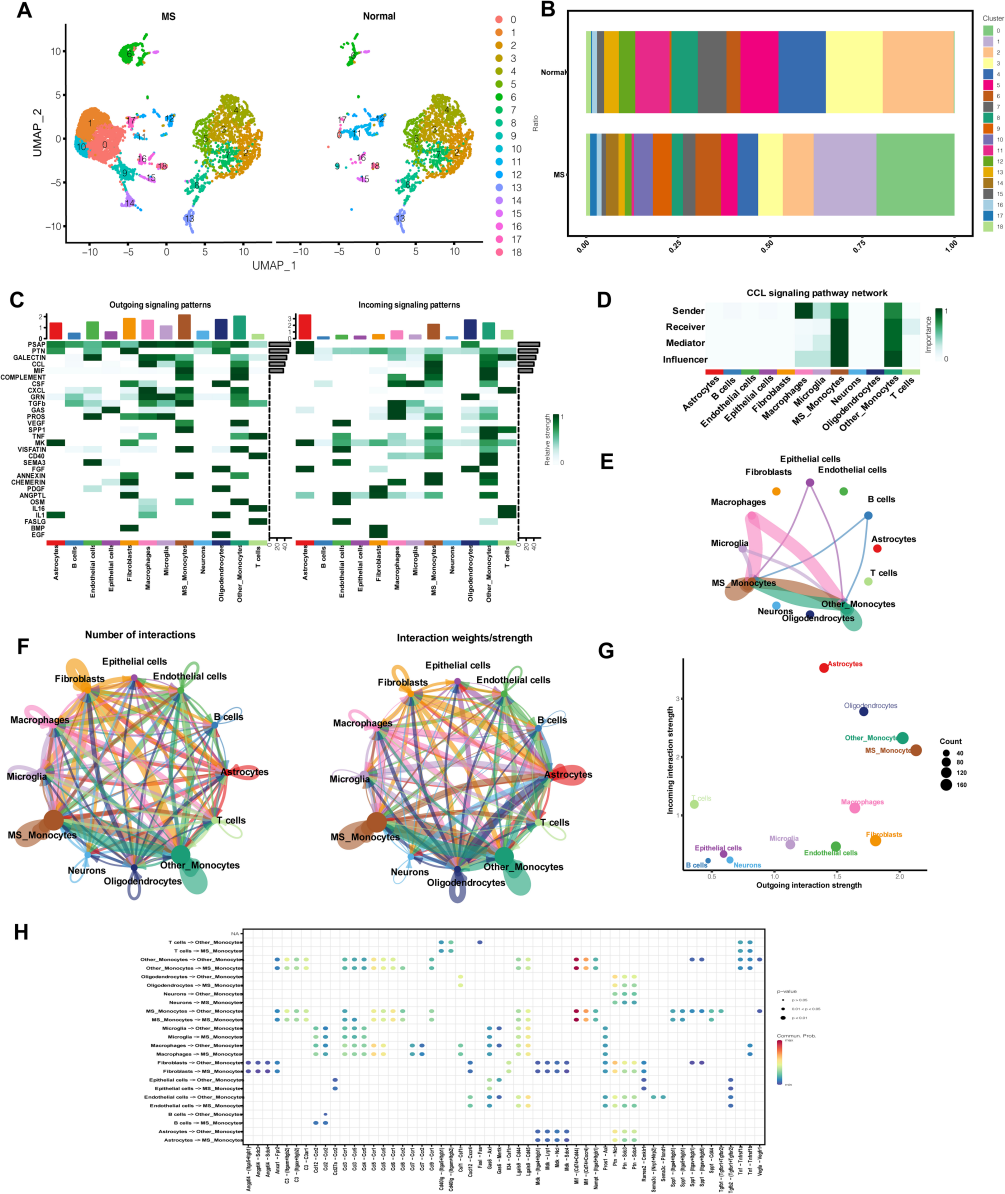
Cellular communication analysis. **(A)** Monocytes were categorized into 18 subpopulations. **(B)** Clusters 0, 1, 9, 10, and 14 were unique to the EAE group. **(C)** The heatmap displays the top cell cytokines. **(D)** Heatmap of bidirectional CCL signaling network between EAE and control groups. **(E)** The circle plot shows the strength of intercellular communication in CCL signaling networks. **(F)** The circle plot shows the number and strength of interactions. **(G)** The scatter plot depicts differences in the strength of incoming and outgoing interactions. **(H)** Comparison of significant intercellular ligand–receptor interactions.

Single-cell sequencing revealed significant differences, implicating monocytes in MS-related pathological changes. To further characterize this population, monocytes were subdivided into 18 subpopulations (Figure 1A). Among these, clusters 0, 1, 9, 10, and 14 were uniquely present in EAE samples, hereafter defined as MS-related monocytes, whereas the remaining clusters were shared with controls and referred to as other-related monocytes (Figure 1B). Intercellular communication was then investigated using the CellChat pipeline. Across all populations, 30 signaling pathways were identified (Figure 1C). Notably, MS-related monocytes exhibited the strongest outgoing interaction strength, suggesting enhanced secretory activity compared to other major cell types (Figures 1F,G). These monocytes also engaged in multiple pro-inflammatory signaling networks, such as CC chemokine ligands (CCL), macrophage migration inhibitory factor (MIF), complement, transforming growth factor (TGF), vascular endothelial growth factor (VEGF), secreted phosphoprotein 1 (SPP1), visfatin, annexin, and oncostatin-M. Further analysis revealed that MS-related monocytes received increased macrophage-derived signals via the CCL pathway (Figures 1D,E). Ligand–receptor interaction mapping showed that macrophages/MS-related monocytes and endothelial cells/MS-related monocytes communicated most strongly through the CCL5–CCR1 and CCL5–CCR5 axes. Additionally, MS-related monocytes displayed a greater number of ligand-receptor pairs with other cell types compared to controls (Figure 1H).

### HdWGCNA analysis highlights modules characterizing the potential functions of MS-related monocytes

**Figure 2:**
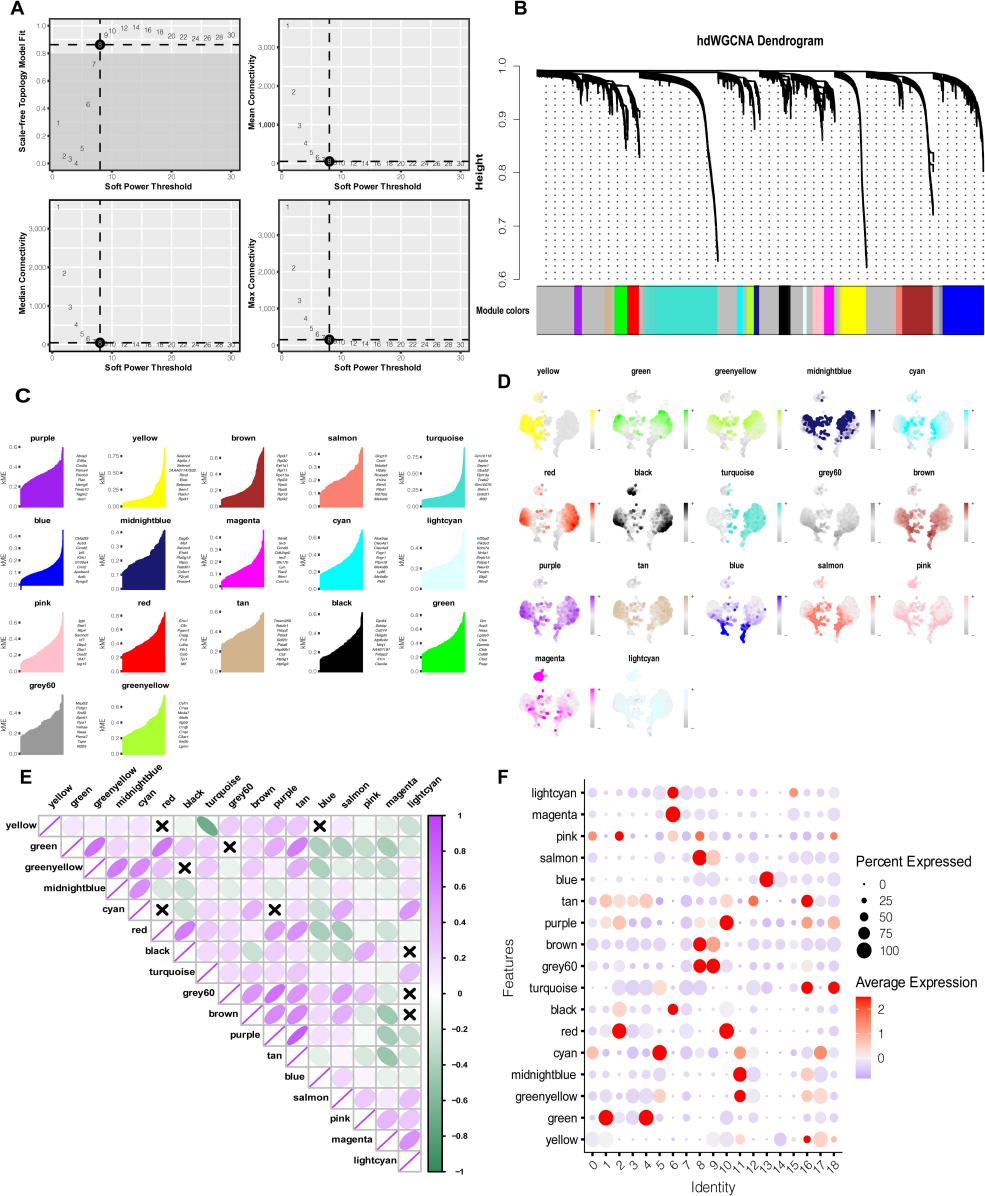
HdWGCNA analysis highlights modules characterizing the potential functions of MS-related monocytes. **(A)** The scale-free topology plots show that 8 was the appropriate soft threshold. **(B)** The components of the co-expression network. **(C)** The top 10 hub genes in each module according to the kME value. **(D)** UMAP plot showing the expression of each color module in monocytes. **(E)** Correlation analysis among modules of different colors. **(F)** HdWGCNA-estimated modular activity in distinct clusters of monocyte subpopulations.

HdWGCNA analysis yielded 17 distinct gene modules. From each module, the top 10 hub genes were selected based on their high module eigengene connectivity (kME)(Figures 2A–C). Expression patterns of selected modules in monocytes were visualized across cellular subpopulations and assessed through gene set enrichment scores (Figure 2D). The correlation functions of different modules highlighted functional similarities among several color modules (Figure 2E).

Module activity was further compared across monocyte subpopulations. Bubble plot analysis revealed that the pink, cyan, green, grey60, brown, red, and purple modules were most strongly associated with clusters 0, 1, 9, 10, and 14—the MS-related monocyte subpopulations (Figure 2F). Collectively, the 70 hub genes identified from these modules were closely linked with MS-related monocyte function and may represent key molecular drivers of disease pathology.

### Trajectory analysis of monocytes reveals candidate gene signatures

Using the differential gene test function, Monocle revealed distinct gene expression changes across monocyte developmental trajectories. Pseudotime progression was ordered from early (light) to late (dark) states. The trajectory profile was divided into three states (Supplementary Figures S3A–C). MS-related monocyte subpopulations were distributed across different developmental stages. Notably, clusters 9 and 14 localized to the early and intermediate stages of monocyte development, clusters 0 and 1 to the intermediate and late stages, and cluster 10 to the transition point between intermediate and late stages (Supplementary Figure S3D). In parallel, the 70 hub genes were grouped into six sets, each enriched at distinct pseudotime intervals of monocyte proliferation (Supplementary Figure S3E). These findings suggest that hub genes regulate monocyte development at specific stages, thereby influencing pathological alterations associated with MS.

### Functional enrichment of candidate characterization genes

**Figure 3:**
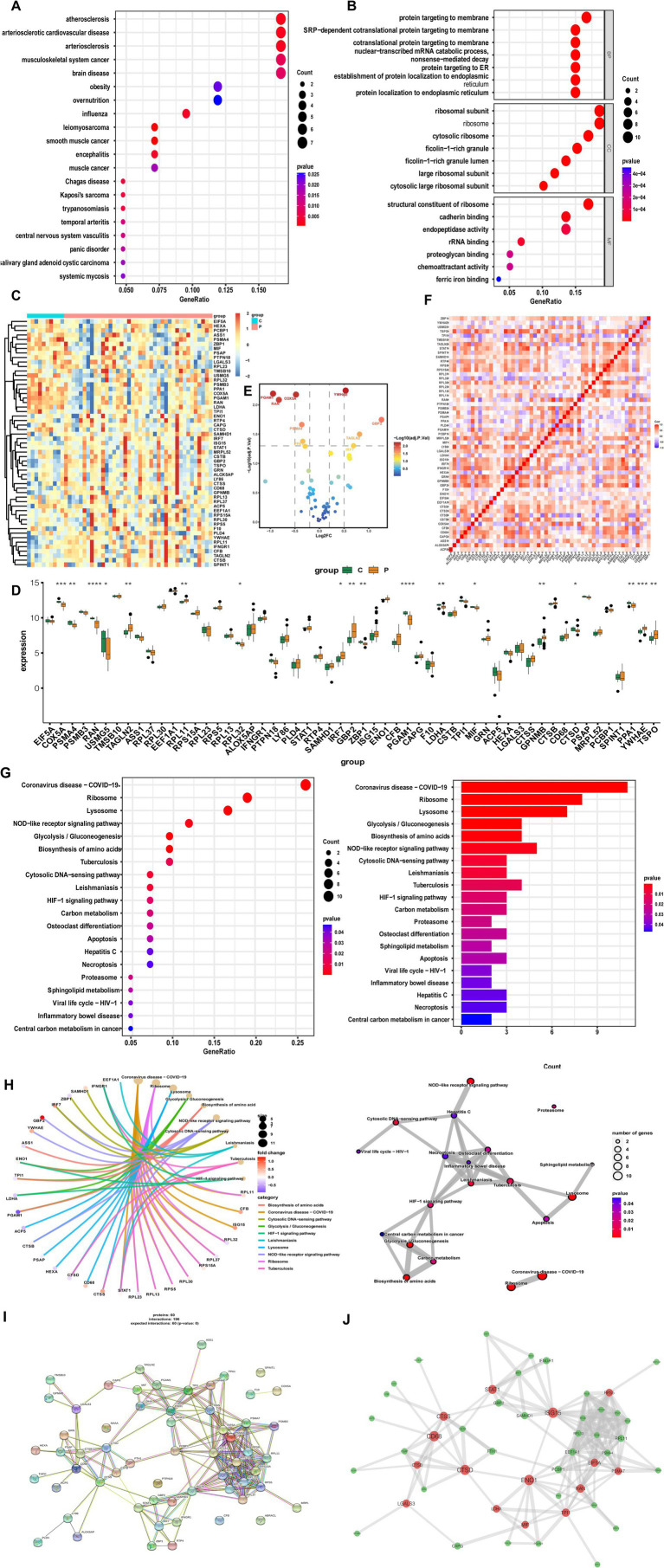
Functional analysis of candidate differentially expressed genes in RNA-sequencing. **(A)** Disease ontology enrichment analysis. **(B)** Gene ontology analysis. **(C)** Heatmap of candidate differential genes in MS and controls. **(D)** Bar graphs showing expression of candidate differential genes in MS and controls. **(E)** Volcano plot of significantly upregulated and downregulated genes. **(F)** Correlation analysis of candidate genes. **(G,H)** Kyoto encyclopedia of genes and genomes analysis. **(I)** Protein–protein interaction network of candidate genes. **(J)** Co-expression network showing correlation strengths among candidate genes.

DO category analysis revealed significant associations of candidate genes with cardiovascular disease, musculoskeletal system cancers, and brain disease (Figure 3A). GO enrichment analysis indicated that hub genes were strongly linked to immune-related processes, such as the ribosomal subunit, ribosome, cytosolic ribosome, and structural constituent of ribosome (Figure 3B). Since ribosomes facilitate translation in immune cells, these findings suggest that ribosomal activity contributes to cellular communication and immune responses.

To further validate hub genes in bulk RNA-seq data, we analyzed transcriptomic profiles from the GSE135511 dataset and identified 55 candidate characterization genes. The bulk RNA-seq analysis was conducted not as a validation of cell type-specific expression in human monocytes, but as an initial, tissue-level correlative study. Its objective was to examine whether the key molecular features identified in the pathological mouse MS-related monocyte state were also reflected in the global transcriptomic profile of lesioned tissue from human MS patients. This approach aimed at establishing a molecular bridge from a defined cellular state to the complex diseased tissue environment, rather than proving cell-type specificity. Heatmap visualization demonstrated clear expression differences between MS and control samples (Figure 3C). Among them, *COX5A*, *PSMA4*, *RAN*, *TAGLN2*, *RPL11*, *GBP2*, *PGAM1*, *LDHA*, *CTSS*, *GPNMB*, *PPA1*, *YWHAE*, and *TSPO* were most significantly differentially expressed (Figure 3D). Overall, 31 genes were upregulated, with *YWHAE*, *GBP2*, *TAGLN2*, and *IRF7* showing the strongest increases, while 24 genes were downregulated, such as *PGAM1*, *COX5A*, and *RAN* (Figure 3E). Correlation analysis revealed strong positive interactions among the 55 candidate genes, suggesting shared biological functions (Figure 3F). KEGG enrichment analysis further demonstrated that these genes were enriched in pathways such as glycolysis/gluconeogenesis, biosynthesis of amino acids, NOD-like receptor signaling, and cytosolic DNA-sensing (Figures 3G,H). Construction of a PPI network provided additional insights into their functional interconnections (Figures 3I,J). Collectively, these results indicate that candidate characterization genes actively shape the autoimmune environment across multiple immune system dimensions.

### Machine learning algorithms to construct MS classification models and evaluate optimally characterized genes

**Figure 4:**
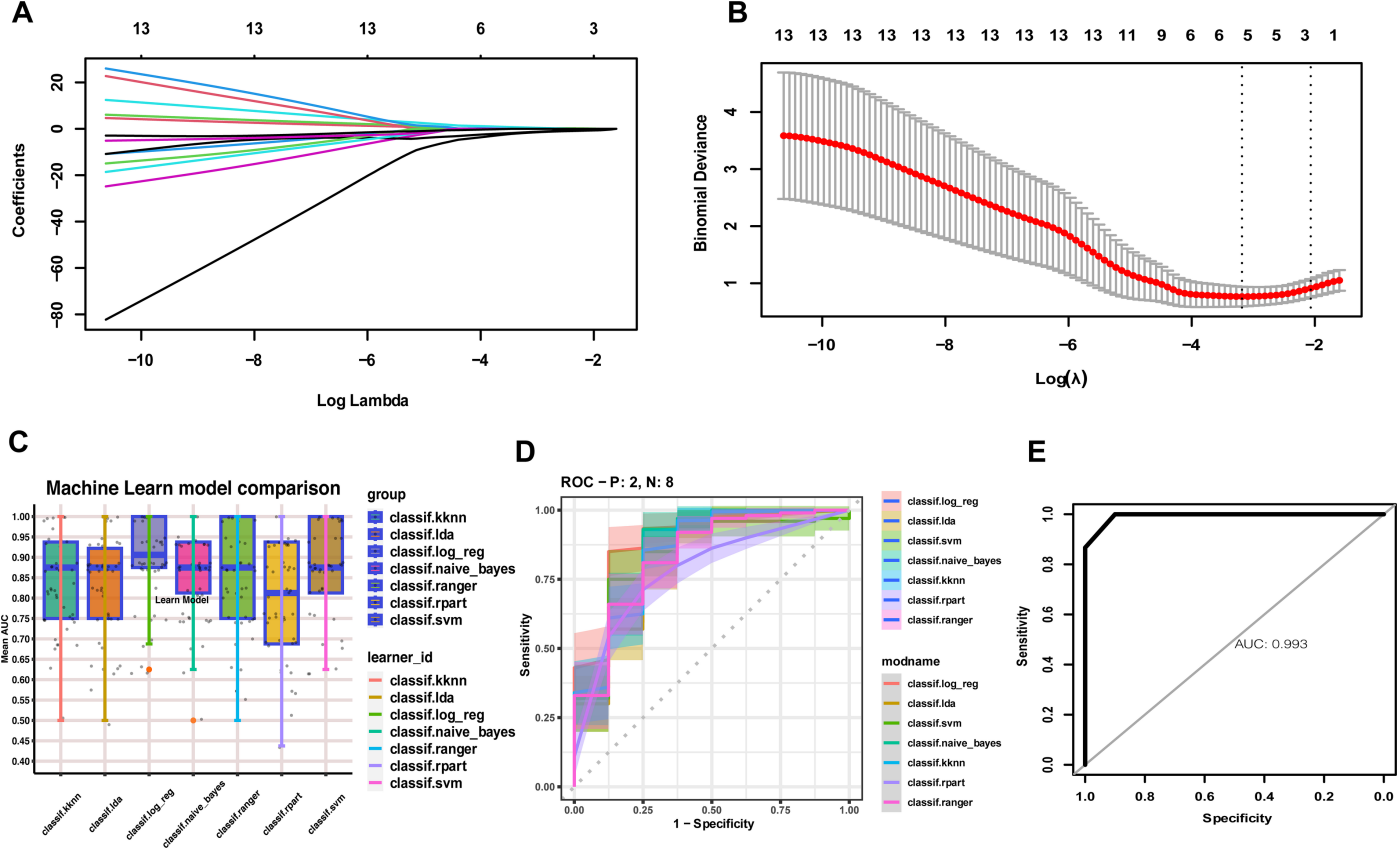
Construction of MS diagnostic models using seven machine learning algorithms. **(A)** LASSO coefficient profiles of the optimally characterized genes. **(B)** Ten-fold cross-validation for parameter selection in the LASSO model. **(C)** Comparison of seven machine learning algorithms for constructing diagnostic models based on five optimally characterized genes. **(D)** ROC curve evaluation of the seven models, with Random Forest performing best in internal validation. **(E)** Random Forest validated in external cohorts.

From the 55 candidate characterization genes, the LASSO algorithm was applied to select the most robust predictors. Five genes—*COX5A*, *CTSS*, *GBP2*, *IRF7*, and *PGAM1*—were retained as optimally characterized candidate biomarkers (Figures 4A,B). Using these five genes, we constructed classification models for MS with seven machine learning algorithms. Model performance was evaluated across cohorts (Figure 4C). Among the classifiers, the RF algorithm demonstrated superior predictive performance in both internal and external validation cohorts (Figures 4D,E).

### Evaluation of the expression and diagnostic importance of optimally characterized genes

We next assessed the expression patterns of the five optimally characterized genes—*COX5A*, *CTSS*, *GBP2*, *IRF7*, and *PGAM1*. Expression analysis revealed that *CTSS*, *GBP2*, and *IRF7* were significantly upregulated in MS samples, whereas *COX5A* and *PGAM1* were markedly downregulated (Supplementary Figures S4A–E). To evaluate discriminative ability, ROC curve analysis was performed. The AUC values were COX5A: 0.091, CTSS: 0.667, GBP2: 0.811, IRF7: 0.755, and PGAM1: 0.904 (Supplementary Figures S4F–J), indicating strong discriminative ability for several of these genes, particularly *GBP2*, *IRF7*, and *PGAM1*.

To ensure reliability, we further validated gene expression levels in an independent external dataset. Consistently, *CTSS*, *GBP2*, and *IRF7* were significantly upregulated, while *COX5A* and *PGAM1* were significantly downregulated in MS samples (Supplementary Figures S5A–E). ROC analysis yielded AUC values of COX5A: 0.957, CTSS: 0.700, GBP2: 0.953, IRF7: 0.673, and PGAM1: 0.780 (Supplementary Figures S5F–J). These validation results further support the potential diagnostic relevance of all five genes in MS.

### TF network analysis identified the potential regulatory regulons

**Figure 5:**
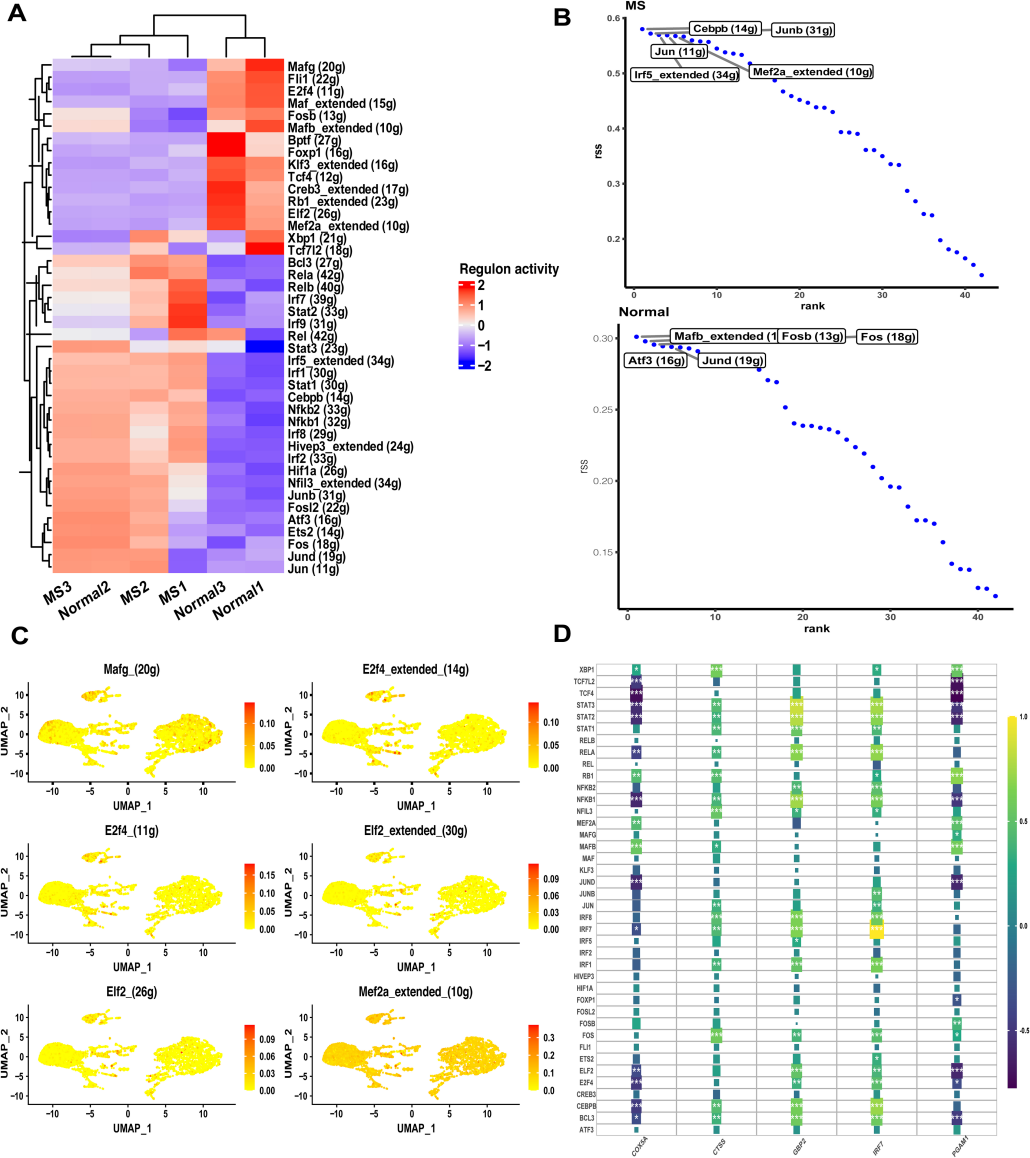
Transcriptional regulatory analyses in MS samples. **(A)** Heatmap showing differences in TF expression between control and MS samples. **(B)** Regulon specificity scores ranking the top TFs in control and MS samples. **(C)** Expression distribution of significantly altered TFs in monocytes. **(D)** Correlation analysis between SCENIC-identified regulons and optimally characterized genes in MS bulk RNA-seq data.

Since TFs regulate gene expression by binding to DNA sequences, we applied the SCENIC pipeline to investigate transcriptional regulatory differences between MS and control samples. The analysis revealed substantial discrepancies in TF activity across the two groups (Figure 5A). Based on the calculated regulon specificity scores, several TFs displayed distinct patterns, such as Cebpb, Junb, Jun, Irf5, and Mef2a (Figure 5B). Among these, Mef2a exhibited higher mRNA expression levels and stronger inferred activity in monocytes compared to other differential TFs (Figure 5C). These TFs are known to play critical roles in immune-related pathways, providing a preliminary overview of cell-type-specific regulators implicated in MS pathology. We next examined associations between divergent TFs and the optimally characterized genes (Figure 5D). The analysis identified significant connections involving STAT, Mef2a, and CEBPB, all of which were correlated with the optimally characterized gene set. Notably, GBP2 demonstrated the strongest overall connectivity with divergent TFs among the five candidate genes. These findings suggest that specific TFs exert multifaceted regulatory effects across monocyte subtypes, potentially contributing to the observed heterogeneity of MS-related monocytes.

### Immune infiltration environment for optimally characterized genes

**Figure 6:**
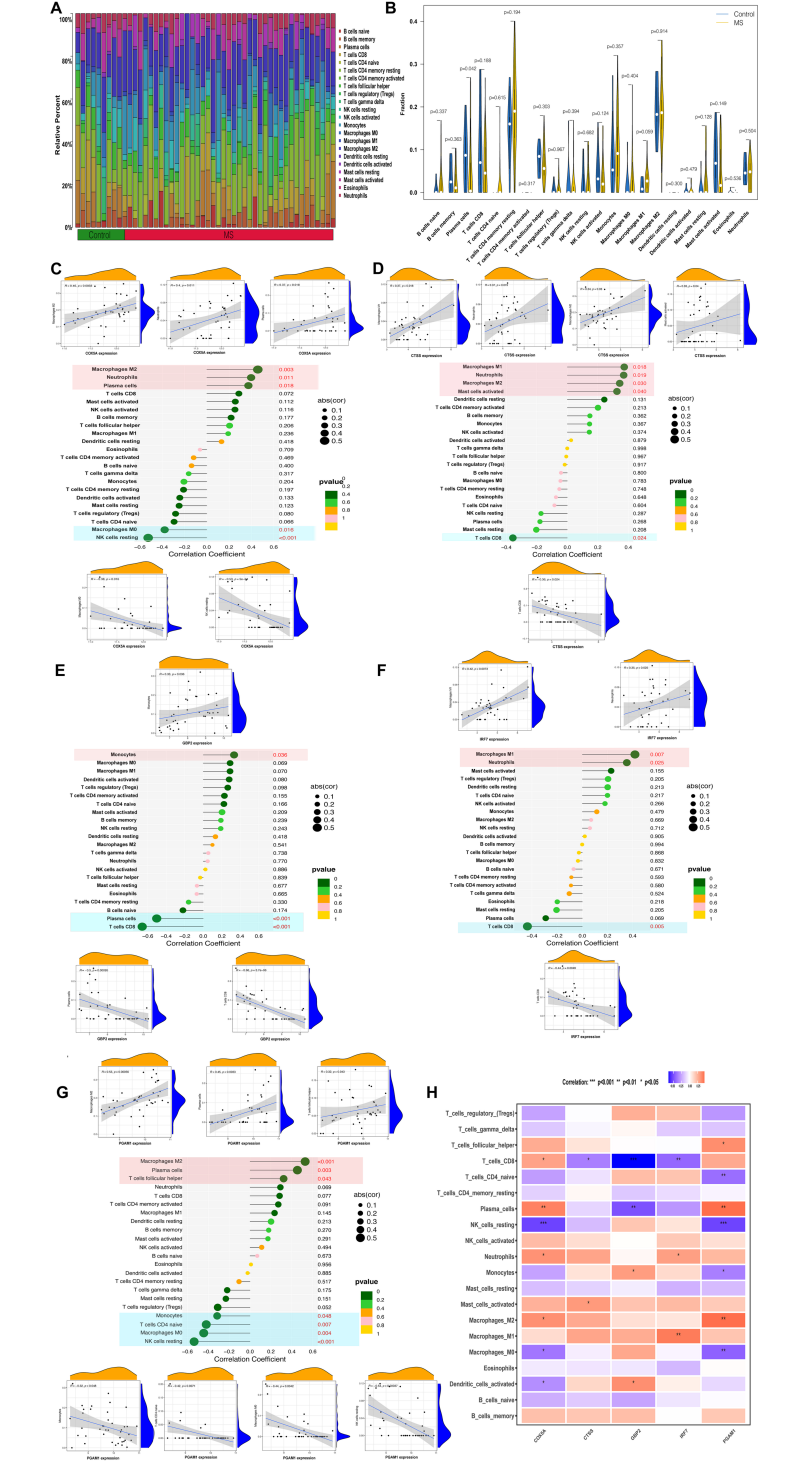
Immune cell infiltration analysis by CIBERSORT. **(A)** The proportions of 22 different types of immune cells in control and MS samples. **(B)** Representative boxplot showing the difference in immune cell infiltration between control and MS samples. **(C–G)** Correlation between immune cells and optimally characterized genes COX5A **(C)**, CTSS **(D)**, GBP2 **(E)**, IRF7 **(F)**, and PGAM1 **(G)**. **(H)** Heatmap showing the correlation of optimally characterized genes with immune cells.

To assess differences in immune cell infiltration between MS and control samples, the CIBERSORT algorithm was applied to the GSE135511 dataset. The proportional distributions of 22 immune cell types in MS and control groups are shown in Figures 6A,B. Correlation analysis was then performed between optimally characterized genes and immune cell subsets (Figures 6C–G). Macrophages M2 were positively correlated with COX5A, CTSS, and PGAM1, whereas neutrophils showed positive correlations with COX5A, CTSS, and IRF7. In contrast, resting NK cells were negatively correlated with COX5A and PGAM1, and CD8^+^ T cells negatively correlated with CTSS, GBP2, and IRF7. Notably, monocytes displayed a positive correlation with GBP2 and a negative correlation with PGAM1 (Figure 6H). These results are consistent with our earlier identification of hub genes linked to MS-related monocyte populations.

### ssGSEA analysis of hallmark gene sets

To compare hallmark pathway activity between MS and control samples, we applied the ssGSEA algorithm to evaluate enrichment across 50 hallmark gene sets. Multiple pathways were significantly different between groups, such as fatty acid metabolism, allograft rejection, peroxisome, IL2–STAT5 signaling, P53 pathway, xenobiotic metabolism, inflammatory response, MYC targets V1/V2, interferon-*γ* response, TGF-*β* signaling, and TNF*α* signaling via NF-κB (Supplementary Figure S6A). These hallmark gene sets may be aberrantly activated and contribute to MS progression. We next examined associations between the five optimally characterized genes and hallmark pathways (Supplementary Figure S6B). CTSS, GBP2, and IRF7 exhibited functional similarities, being enriched in pathways such as xenobiotic metabolism, P53 signaling, interferon-γ response, interferon-α response, inflammatory response, IL6-JAK-STAT3 signaling, and IL2–STAT5 signaling. In contrast, COX5A and PGAM1 shared enrichment in pathways such as oxidative phosphorylation and fatty acid metabolism. Collectively, these results suggest that the optimally characterized genes exert their effects through distinct but complementary hallmark pathways, providing potential mechanistic insights into their contribution to immune dysregulation in MS.

### Functional identification of optimally characterized genes

Given the strong diagnostic significance of the five optimally characterized genes in MS progression and immune regulation, we next applied the GSEA algorithm to investigate their potential physiological functions. For COX5A, the high-expression subgroup was significantly enriched in pathways, such as oxidative phosphorylation and retrograde endocannabinoid signaling (Supplementary Figure S7A). Conversely, the low-expression subgroup showed enrichment in ECM–receptor interaction, glycosaminoglycan biosynthesis (chondroitin sulfate/dermatan sulfate), and vitamin digestion/absorption (Supplementary Figure S8A). For GBP2, high expression was associated with allograft rejection and systemic lupus erythematosus (Supplementary Figure S7B), while low expression was enriched in glycosaminoglycan biosynthesis (heparan sulfate/heparin) and synaptic vesicle cycle (Supplementary Figure S8B). For IRF7, the high-expression subgroup was enriched in antifolate resistance, legionellosis, and systemic lupus erythematosus (Supplementary Figure S7C). In contrast, low expression was enriched in basal TFs, endocrine-regulated calcium reabsorption, and glycosaminoglycan biosynthesis (heparan sulfate/heparin) (Supplementary Figure S8C). For PGAM1, high expression was associated with butanoate metabolism, nicotine addiction, oxidative phosphorylation, protein export, and type I diabetes mellitus (Supplementary Figure S7D), whereas low expression was enriched in ABC transporters and fat digestion/absorption (Supplementary Figure S8D). For CTSS, the high-expression subgroup was enriched in allograft rejection, graft-versus-host disease, legionellosis, leishmaniasis, and type I diabetes mellitus (Supplementary Figure S7E), while low expression was associated with ABC transporters, *α*-linolenic acid metabolism, linoleic acid metabolism, retrograde endocannabinoid signaling, and vitamin digestion/absorption (Supplementary Figure S8E). Notably, butanoate metabolism and oxidative phosphorylation emerged as key pathways in the high-expression groups of COX5A and PGAM1, while systemic lupus erythematosus and leishmaniasis were dominant in the high-expression groups of GBP2 and IRF7. Correlation analysis further demonstrated strong positive associations among the five genes (Supplementary Figure S9A,B). The correlation between PGAM1 and COX5A was particularly high (*r* = 0.90), while CTSS, GBP2, and IRF7 exhibited marked functional similarities, supporting their cooperative role in MS pathogenesis.

### Identification of optimally characterized genes

**Figure 7:**
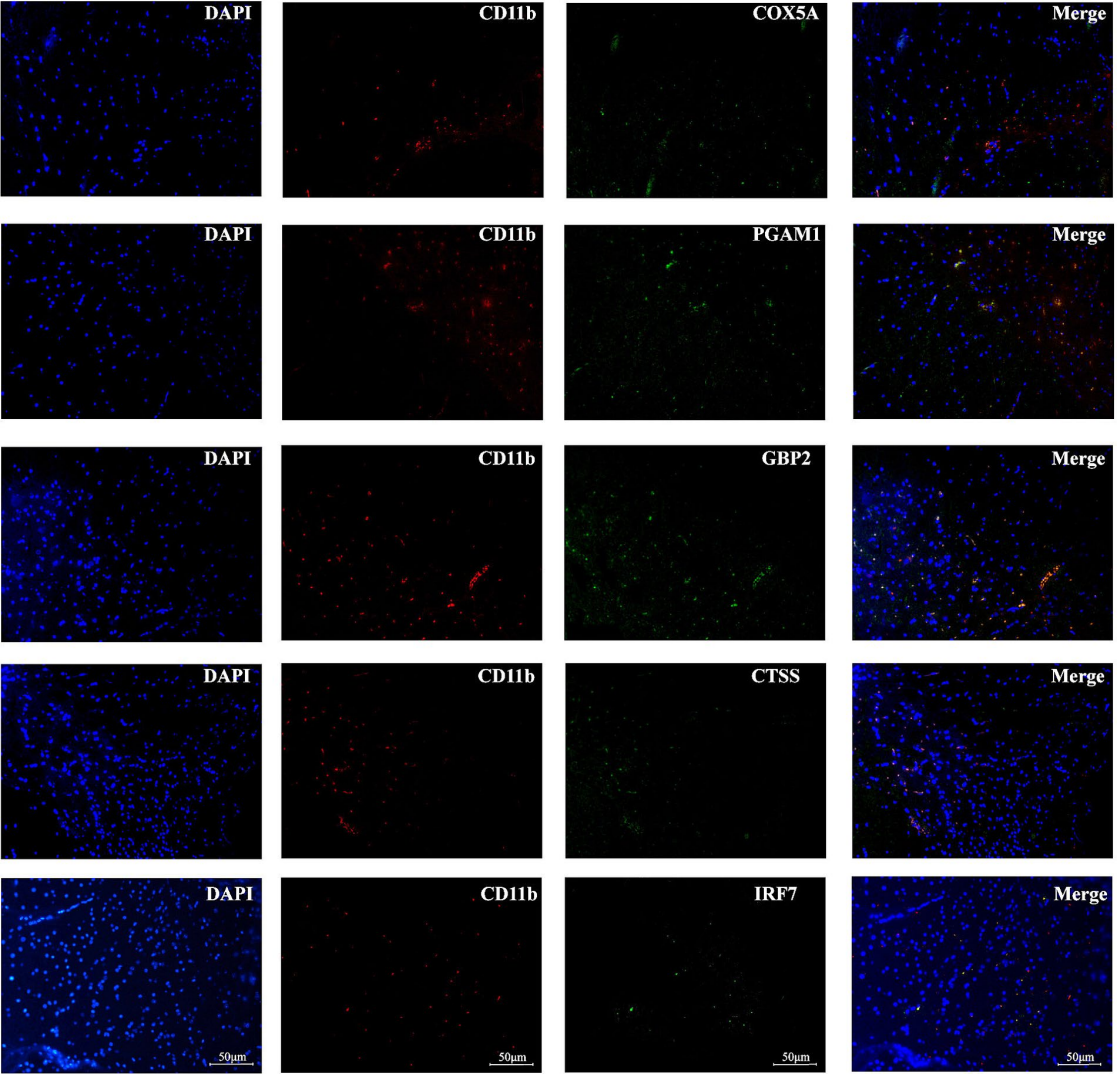
QRT-PCR validation of five candidate genes. Expression levels in three pairs of EAE and control brain tissues are shown as mean ± SE (*p* < 0.05).

The expression levels of the five optimally characterized genes—*COX5A*, *CTSS*, *GBP2*, *IRF7*, and *PGAM1*—were further validated by qRT-PCR (Figure 7). The qRT-PCR results were consistent with the microarray findings. Specifically, *CTSS*, *GBP2*, and *IRF7* were significantly upregulated in the brain tissue of EAE samples, whereas *COX5A* and *PGAM1* were markedly downregulated (*p* < 0.05).

### Optimally characterized genes localize to monocytes in brain tissues after EAE

**Figure 8:**
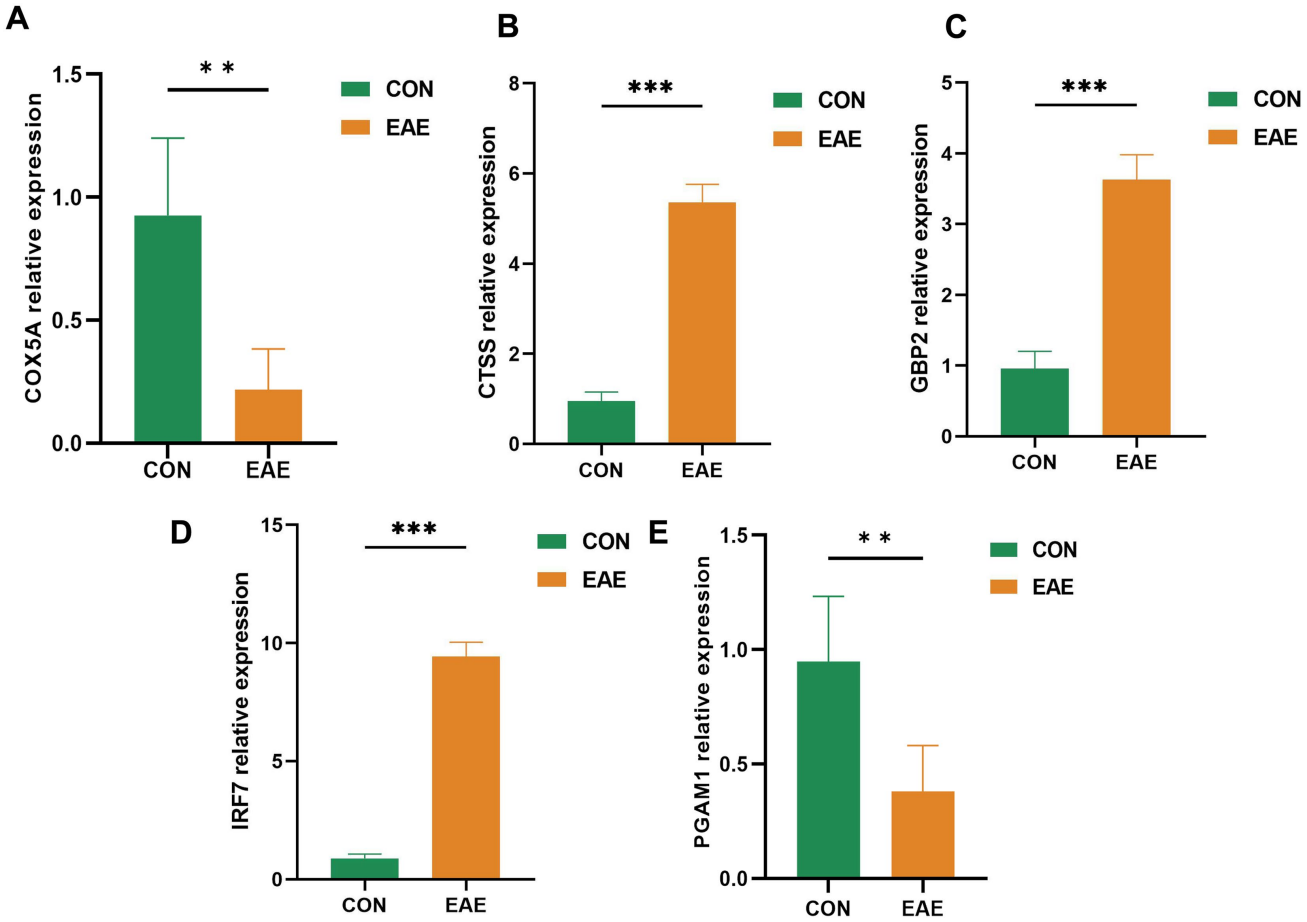
Immunofluorescence analysis of optimally characterized genes. COX5A, CTSS, GBP2, IRF7, and PGAM1 (green fluorescence) were observed in CD11b immunopositive myeloid cells of brain tissues after immune induction. Nuclei are counterstained with DAPI (blue signal). Scale bars = 50 mm.

Immunofluorescence analysis confirmed the localization of the five optimally characterized genes—*COX5A*, *CTSS*, *GBP2*, *IRF7*, and *PGAM1*—within monocytes in brain tissues following immune induction (Figure 8). Among these, GBP2 showed the most prominent expression, consistent with the results of bioinformatic analyses. Representative immunofluorescence staining further demonstrated that the optimally characterized genes were expressed in CD11b^+^ immunopositive myeloid cells.

## Discussion

This study identified a functionally active and significantly expanded subset of MS-associated monocytes in EAE brain tissue through single-cell sequencing. In EAE, Ly6c2 is recognized as the most definitive and stable lineage marker for classical inflammatory monocytes, which are characterized by a Ly6C-high, CCR2-dependent phenotype. Its expression profile inherently validates the identity of the CCR2^+^ monocyte subset (30, 31). Chil3 functions as an auxiliary pathological marker, being selectively upregulated within neuroinflammatory lesions by activated myeloid cells, such as those of monocytic origin. This expression pattern robustly signifies a disease-associated activation state, offering greater discriminatory power for identifying pathology-specific phenotypes compared to Fcgr1, which is ubiquitously expressed across monocyte and macrophage populations and exhibits limited specificity in this context (32, 33). Collectively, the combined use of Ly6c2 and Chil3 establishes a rigorous and sufficient basis for the precise identification of this monocyte population within the EAE pathological framework. Integrated analysis of cell–cell communication and pseudotime trajectory revealed that this population undergoes dynamic differentiation within the inflammatory microenvironment and serves as an immune signaling hub, largely mediated through axes such as CCL5. The MS-related monocytes partially overlap in pro-inflammatory phenotype with the classically described Ly6C^+^CCR2^+^ inflammatory monocytes, which infiltrate the central nervous system via the CCL2-CCR2 axis and differentiate into pro-inflammatory macrophages, serving as key drivers of neuroinflammation (34). This population also exhibits high expression of chemokine receptors, such as CCR1 and CCR5, along with inflammation-associated genes, such as GBP2, IRF7, and CTSS, indicative of a highly activated state. However, HdWGCNA analysis further revealed unique co-expression modules and distinct metabolic gene signatures (COX5A and PGAM1), suggesting that this group does not represent an entirely novel subtype, but rather a functionally specialized state arising from transcriptional reprogramming and metabolic adaptation of classical inflammatory monocytes within the disease microenvironment. Cell–cell communication and pseudotemporal trajectory analyses demonstrated that MS-related monocytes constitute a dynamic continuum undergoing continuous differentiation and polarization, engaging in close interaction with infiltrating macrophages through signaling axes such as CCL5 (35). We propose that newly recruited monocytes undergo functional reprogramming within the CNS inflammatory milieu, particularly under the guidance of macrophage-derived signals, progressively differentiating into effector cells with potent pro-inflammatory and immunomodulatory capacities. This dynamic adaptability enables the sustained driving of neuroinflammation and may represent a key cellular mechanism underlying the perpetuation and expansion of immune-mediated damage in MS.

Machine learning-based screening identified five optimally characterized genes from this cellular population, which showed excellent diagnostic performance in an independent validation cohort. CTSS is a key regulator of monocyte activation (36). As a lysosomal cysteine protease, CTSS generates peptides that bind to MHC class II molecules, thereby facilitating the activation of CD4^+^ T cell immune responses. This function is supported by evidence from CTSS^−^/^−^mice, which exhibit significantly attenuated EAE symptoms (37). Furthermore, CTSS contributes to blood–brain barrier (BBB) disruption by binding to and degrading junctional adhesion molecule (JAM) family proteins (36). Finally, CTSS can directly upregulate the expression of pro-inflammatory cytokines, such as IL-6 and IL-1*β*, through pathways such as protease-activated receptor (PAR) 2 activation (38), thereby amplifying the local inflammation. Consequently, the elevated expression of CTSS in MS-related monocytes observed in this study likely enhances their antigen-presenting capacity, promotes BBB damage, and augments their pro-inflammatory potential, collectively driving neuroimmunopathological progression.

In the pathological context of MS, IRF7 contributes to BBB disruption by monocytes (39). As a key transcription factor for IFN-*α*/β synthesis, IRF7 is persistently activated and drives the production of IFN-α. The IRF7/IFN-α axis further upregulates the expression of GBP2. Beyond its direct role in host defense, GBP2 potently enhances the phosphorylation and dimerization of STAT1 (40), thereby significantly amplifying the intensity and duration of downstream interferon signaling and establishing a critical positive feedback amplification loop. Additionally, studies have confirmed that GBP2 participates in the assembly and activation of the NLRP3 inflammasome (41), promoting the maturation and release of pyroptosis-related factors such as IL-1β, which directly exacerbates tissue inflammatory damage. The downregulation of COX5A and PGAM1 identified in this study collectively reveals a distinct metabolic reprogramming in MS-related monocytes. PGAM1, a key enzyme in the glycolytic pathway (42), is critically involved in rapid ATP generation and biosynthetic precursor production. The observed transcriptional downregulation of PGAM1 may indicate the presence of post-transcriptional regulation or a stage-specific metabolic adaptation during disease progression. Concurrently, the decreased expression of COX5A, an essential subunit of mitochondrial complex IV of the electron transport chain, clearly suggests a metabolic shift from efficient oxidative phosphorylation toward glycolysis. This metabolic remodeling supplies the necessary energetic substrates and biosynthetic building blocks that support the high-energy-demanding immune activities of monocytes, thereby fundamentally sustaining their activated pathological state. Based on the evidence presented above, the five candidate genes are implicated in the immune dysregulation of MS via multiple interconnected pathways.

During independent validation of the core biomarker genes, notable discrepancies in AUC values were observed between the training set (GSE135511) and the external validation set (GSE108000). Such variability is commonly encountered in studies utilizing public omics data, primarily attributable to inter-cohort heterogeneity in clinical staging, treatment regimens, and sample types, as well as technical differences between microarray platforms (GPL6883 vs. GPL13497). Nevertheless, the key conclusions of this study remain clear, the direction of expression changes for all genes was concordant across both datasets, and the machine learning model consistently demonstrated robust performance in both internal and external validations. Rather than diminishing the significance of our findings, the observed cross-dataset variation underscores the necessity for further validation in larger, prospectively collected clinical cohorts with well-annotated phenotypic data.

Based on publicly available sequencing datasets, this study applied a conceptual and innovative approach to explore MS risk genes with greater precision. Nevertheless, several limitations should be acknowledged. First, the single-cell sequencing results were derived from EAE brain tissue and have not yet been validated in human samples. Second, the EAE model does not fully capture the heterogeneity of MS and cannot classify disease progression into distinct clinical subtypes. A limitation of this study concerns the definition of monocyte identity in the scRNA seq analysis. The cell population was annotated based on automated classification (SingleR) and the expression of myeloid-associated genes (Ly6c2 and Chil3), with the latter reflects a state activated in disease rather than an exclusive monocyte marker. As canonical markers, such as Ccr2, and the broadly expressed Fcgr1 were not visualized, the analyzed cluster is best described as an inflammatory myeloid population enriched in monocytes, which may partially overlap with related subsets. Therefore, downstream analyses (e.g., pseudotime and cell–cell communication) reflect transcriptional features of this disease-associated state rather than a definitive monocyte identity. The interpretation of the human bulk RNA seq data must acknowledge its limitations at the tissue level. Although the identified hub genes are evolutionarily conserved and play roles in innate immunity, this does not confirm their expression is specific to monocytes in human MS. Bulk data cannot attribute expression to specific cell types; changes in ubiquitous metabolic genes (e.g., COX5A, PGAM1) may originate from neurons or glia. Thus, these bulk transcriptomic data provide an exploratory assessment at the tissue level of whether molecular features derived from a myeloid state in murine EAE are also present in human MS lesion pathology. Consistent with this interpretation, the CIBERSORT analysis offers indirect, contextual support by correlating gene expression at the tissue level with inferred immune infiltration, but it does not prove specificity to a cell type or causality. Definitive validation of expression specific to monocytes in human MS remains beyond the scope of this study, as it requires transcriptomics resolved at the single-cell or cell-type level, thereby representing an important direction for future research.

## Conclusion

Analysis of single-cell RNA sequencing data from EAE brain tissue revealed a markedly expanded and functionally distinct monocyte population, designated as MS-related monocytes. These cells function as dynamic signaling hubs within the neuroinflammatory network and exhibit continuous differentiation along a pseudotemporal trajectory. Transcriptomic network analysis further revealed core co-expression modules that link their phenotype to underlying molecular drivers. From these modules, machine learning-based screening identified five key genes (COX5A, CTSS, GBP2, IRF7, PGAM1), which not only demonstrated strong biomarker potential in independent cohorts but also reflect two pivotal dimensions of MS pathology: immune-inflammatory activation mediated through interferon response and antigen presentation, and immunometabolic reprogramming involving oxidative phosphorylation and glycolysis. Collectively, these findings position MS-related monocytes as a pathogenic cellular state that bridges immune dysregulation with metabolic adaptation, offering both novel diagnostic biomarkers and mechanistic insights into sustained neuroinflammation in MS.

## Data Availability

The original contributions presented in the study are included in the article/Supplementary material, further inquiries can be directed to the corresponding author.

## Footnotes

1
https://ms-coalition.org/wp-content/uploads/2019/06/MSC_DMTPaper_062019.pdf

2
https://www.ncbi.nlm.nih.gov/geo/

## Supplementary material

SUPPLEMENTARY FIGURE S1 Cluster analysis of single-cell subtypes. **(A)** Unprocessed single-cell sequencing data. **(B)** 48,146 cells were detected in GSE199460. **(C)** PCA and UMAP plots showing the batch differences in single-cell data. **(D)** Batch effects removed using “harmony". **(E)** UMAP clustering of 26 single-cell clusters. **(F)** Cell lineage annotation. **(G,H)** The relative abundance of each cell type in the EAE **(G)** and control groups **(H)**. **(I)** Comparative analysis of cell type distribution.

SUPPLEMENTARY FIGURE S2 Validation of the canonical monocyte protein markers. **(A)** UMAP visualization of all cell types. **(B)** Dot plot displaying the expression of marker genes for each cell type. **(C)** Expression levels of the signature gene Ly6c2 in monocytes. **(D)** Expression levels of the signature gene Chil3 in monocytes.

SUPPLEMENTARY FIGURE S3 Pseudotime analysis of monocytes. **(A)** Data preprocessing and selection of representative genes. **(B)** The order of pseudotime was from light to profound. **(C)** Three states were calculated by pseudotime analysis. **(D)** Pseudotime distribution of the 15 monocyte clusters. **(E)** The heatmap displays the dynamic expression of 70 hub genes across six pseudotime clusters.

SUPPLEMENTARY FIGURE S4 Comparison of expression levels and diagnostic efficacy of optimally characterized genes in the training set. **(A–E)** CTSS **(B)**, GBP2 **(C)** and IRF7 **(D)** expression levels were significantly up-regulated in the MS patient cohort, and COX5A **(A)** and PGAM1 **(E)** were significantly down-regulated in MS. **(F–J)** ROC curves evaluating the diagnostic performance of COX5A **(F)**, CTSS **(G)**, GBP2 **(H)**, IRF7 **(I)**, PGAM1 **(J)**.

SUPPLEMENTARY FIGURE S5 Comparison of expression levels and diagnostic efficacy of optimally characterized genes in the external validation set. **(A–E)** CTSS **(B)**, GBP2 **(C)** and IRF7 **(D)** expression levels were significantly up-regulated in MS samples, and COX5A **(A)** and PGAM1 **(E)** were significantly down-regulated in MS samples. **(F–J)** ROC curves evaluating the diagnostic performance of COX5A **(F)**, CTSS **(G)**, GBP2 **(H)**, IRF7 **(I)**, and PGAM1 **(J)**.

SUPPLEMENTARY FIGURE S6 Single-sample gene set enrichment analysis of hallmark gene sets. **(A)** Comparison of the distribution of the 50 hallmark gene sets in control and MS samples. **(B)** Correlation analysis of 50 hallmark gene sets with optimally characterized genes.

SUPPLEMENTARY FIGURE S7 GSEA analysis of high-expression subgroups. **(A–E)** Top five significantly enriched pathways in the high-expression subgroups of COX5 **(A)**, GBP2 **(B)**, IRF7 **(C)**, PGAM1 **(D)**, and CTSS **(E)**.

SUPPLEMENTARY FIGURE S8 GSEA analysis of low-expression subgroups. **(A–E)** Top five significantly enriched pathways in the low-expression subgroups of COX5 **(A)**, GBP2 **(B)**, IRF7 **(C)**, PGAM1 **(D)**, and CTSS **(E)**.

SUPPLEMENTARY FIGURE S9 Correlation analysis of optimally characterized genes. **(A,B)** Correlation matrix showing strong positive relationships among the five genes in MS samples.
