# Supplementary figures and images for: Machine learning and single-cell RNA sequencing analyses identify MS-related monocytes and a five-gene candidate biomarker signature
Source: Front Neurol. 2026 Feb 11;17:1739231. doi: 10.3389/fneur.2026.1739231 (PMC12932200; doi:10.3389/fneur.2026.1739231)

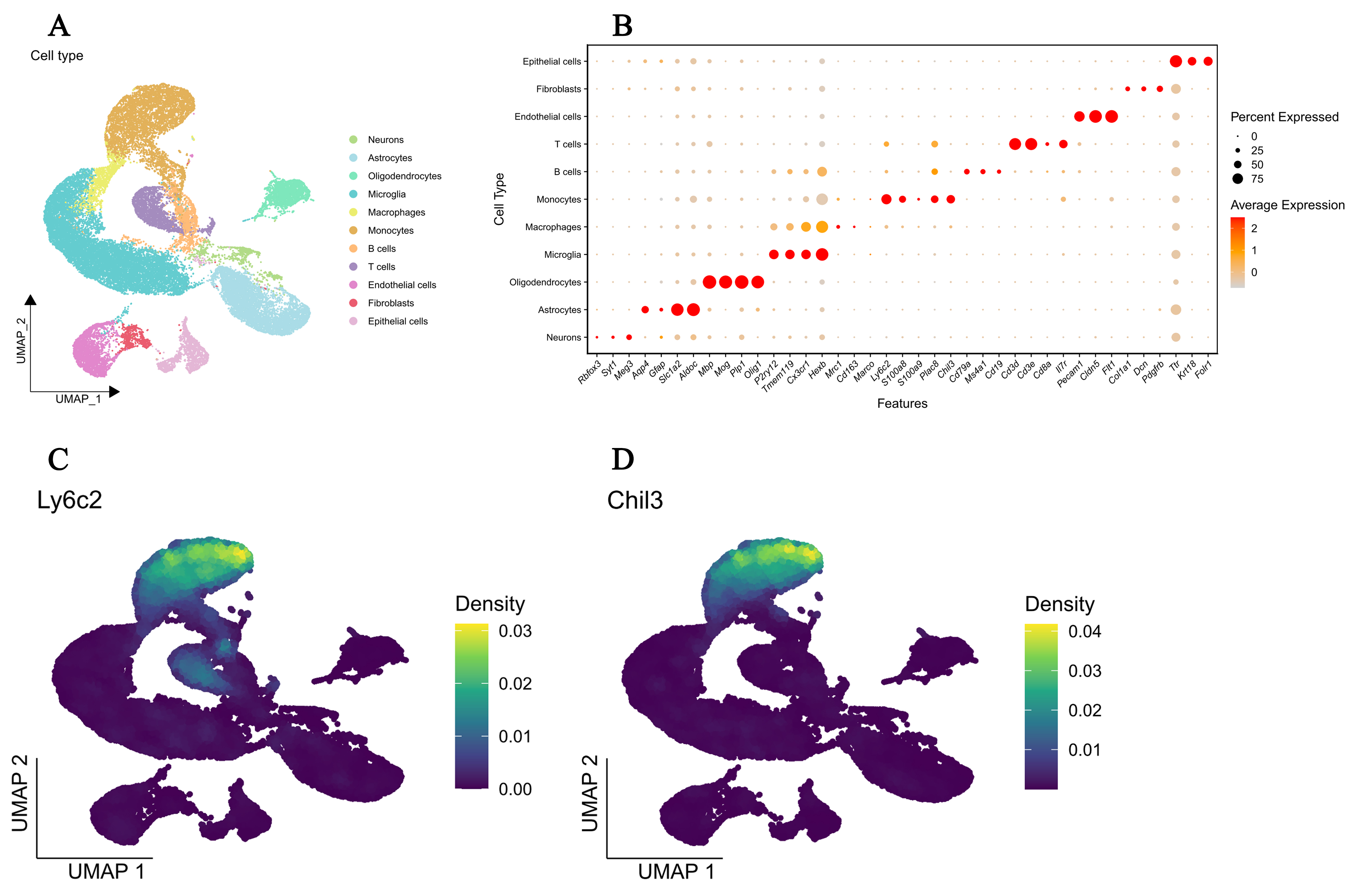

Supplement: Supplementary file 2 [file Image_2.JPEG]

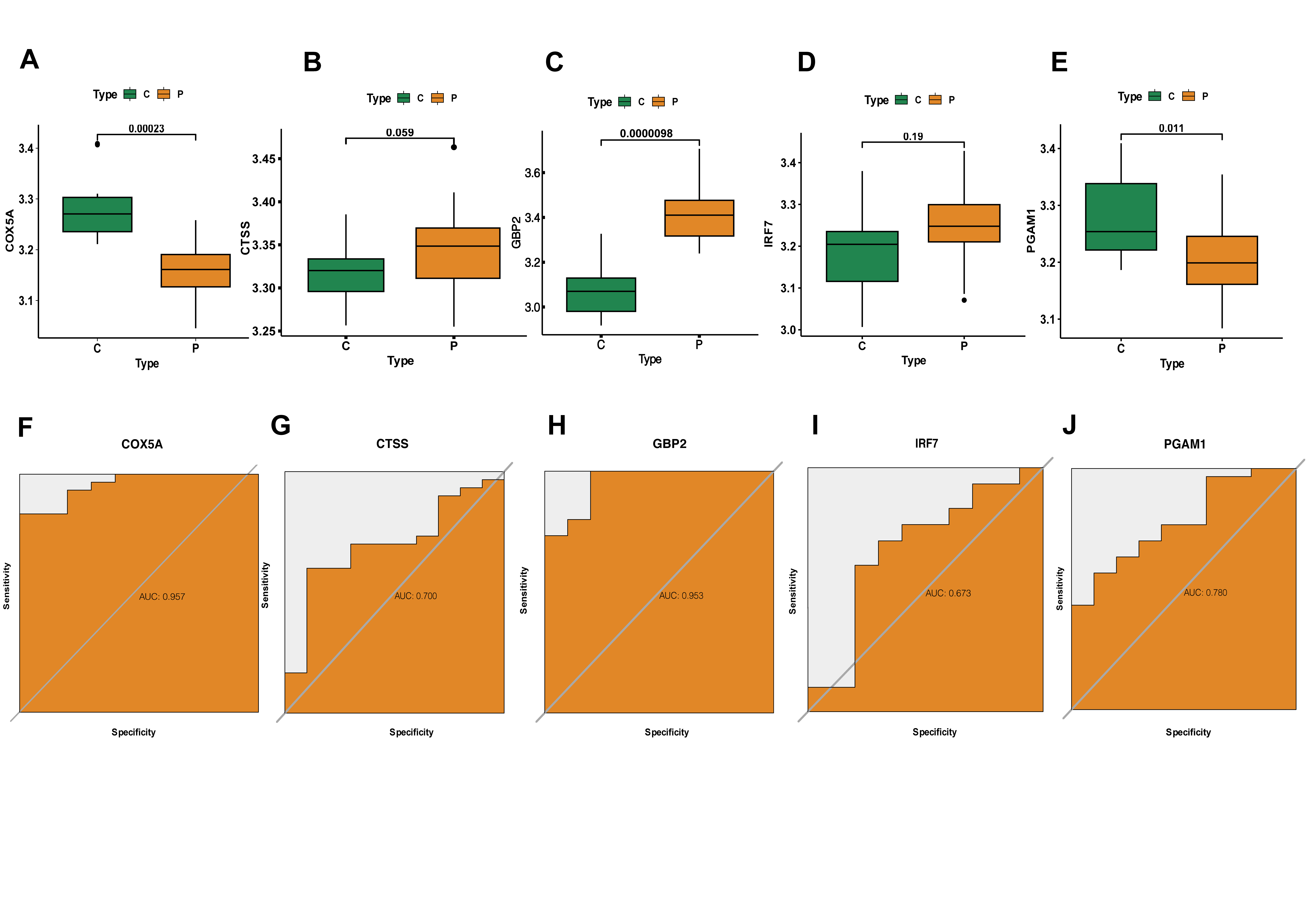

Supplement: Supplementary file 5 [file Image_5.JPEG]

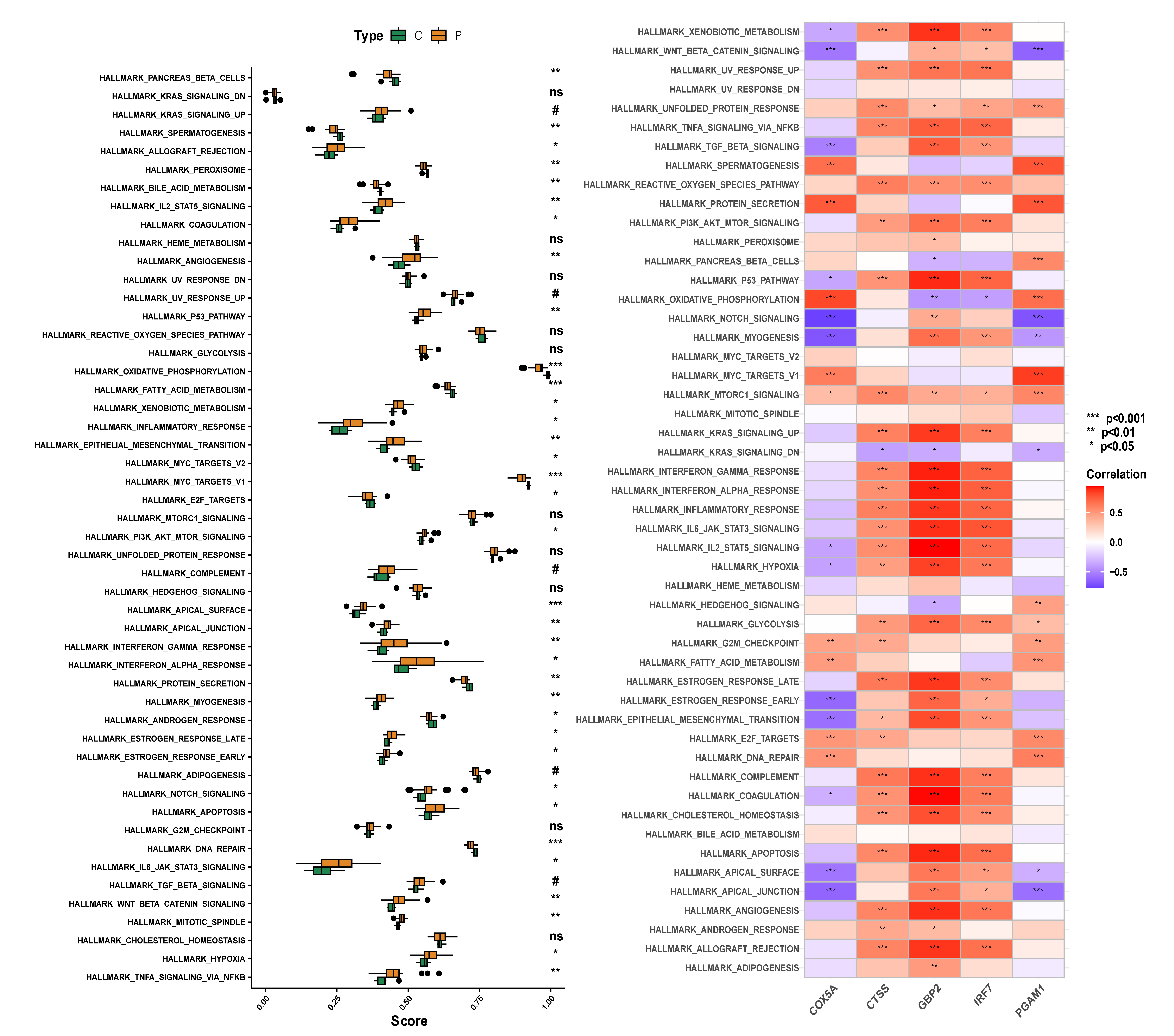

Supplement: Supplementary file 6 [file Image_6.JPEG]

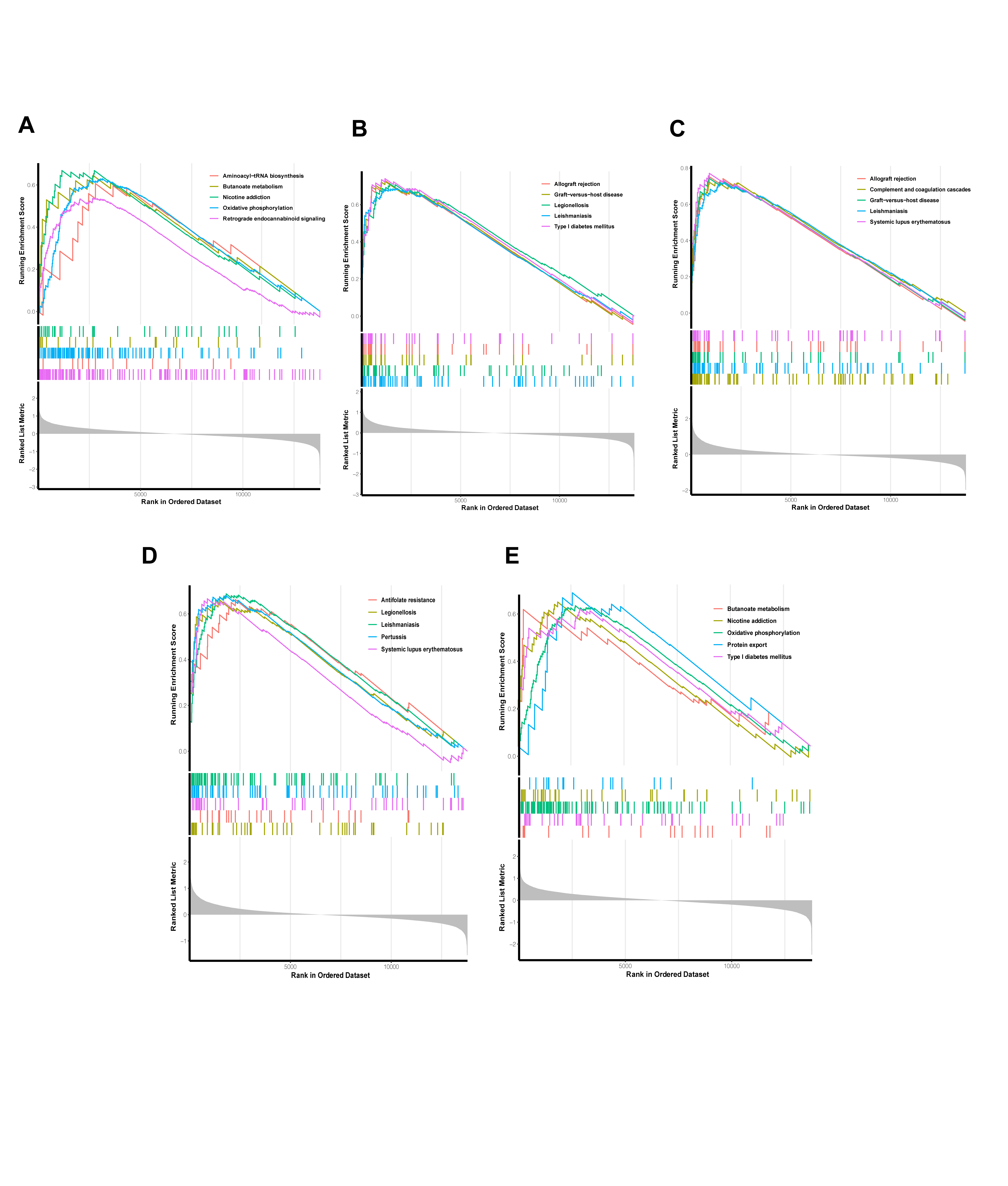

Supplement: Supplementary file 7 [file Image_7.JPEG]

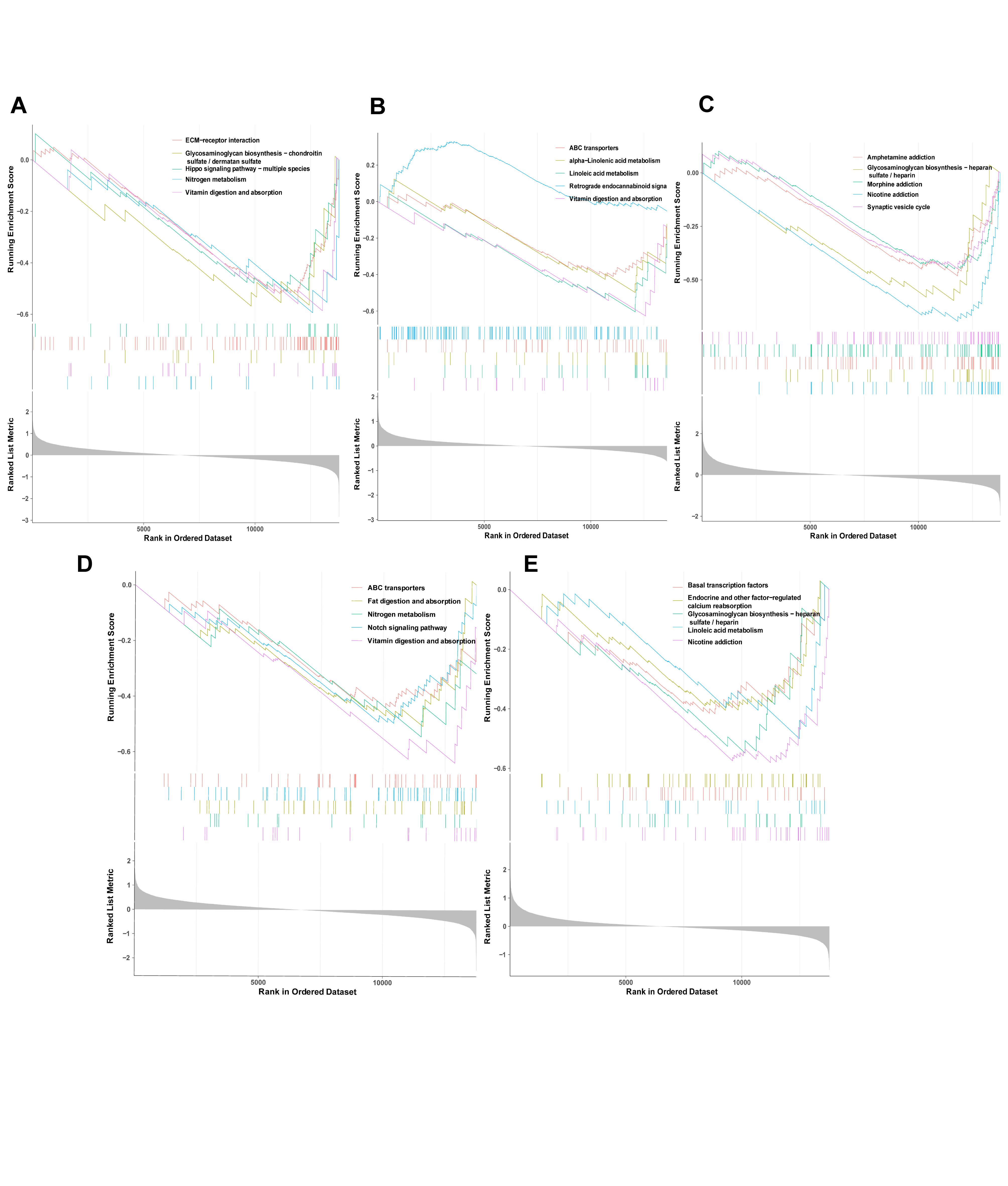

Supplement: Supplementary file 8 [file Image_8.JPEG]

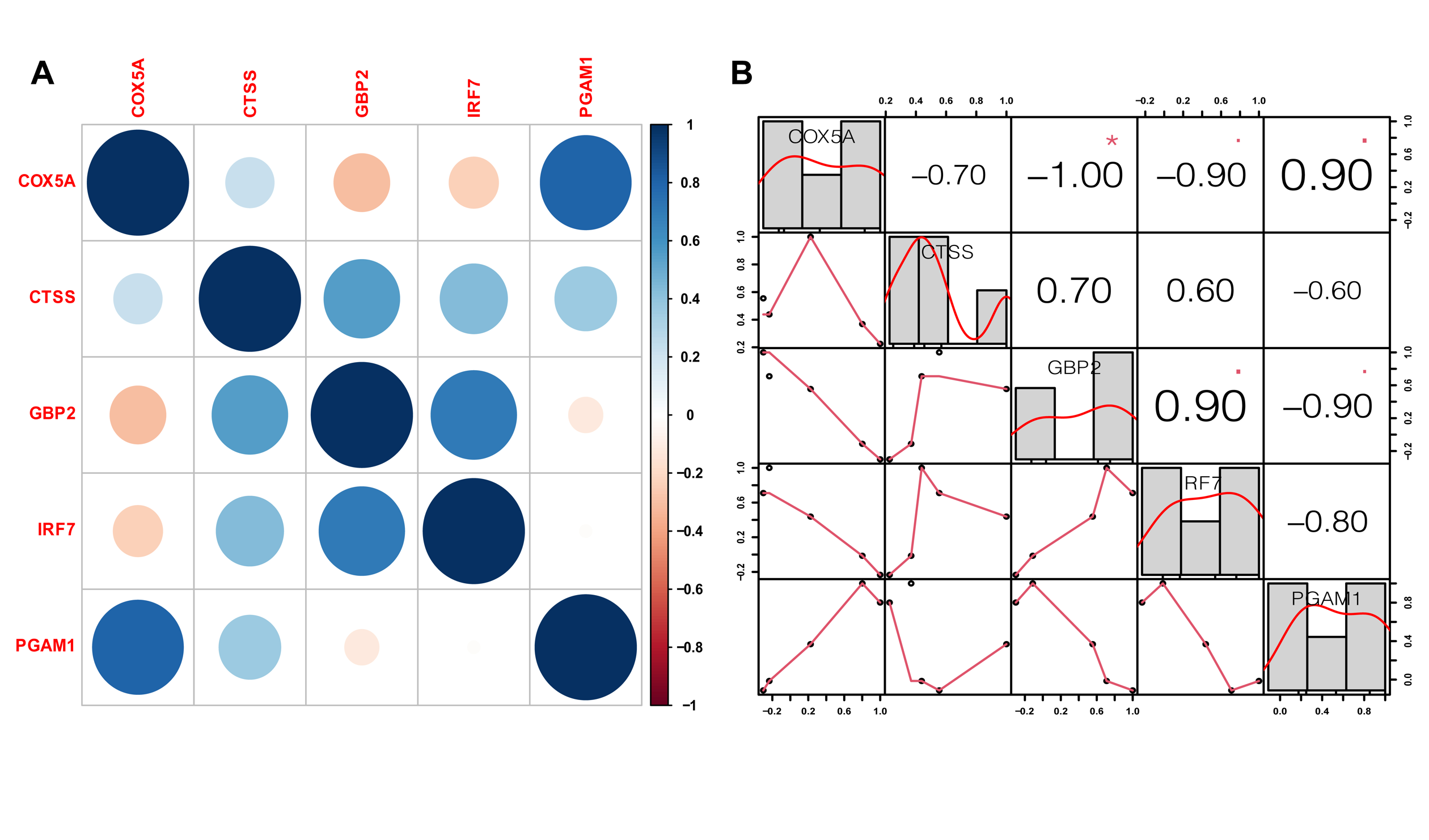

Supplement: Supplementary file 9 [file Image_9.JPEG]
